# Supplementary material for: Content-rich biological network constructed by mining PubMed abstracts
Source: BMC Bioinformatics. 2004 Oct 8;5:147. doi: 10.1186/1471-2105-5-147 (PMC528731; doi:10.1186/1471-2105-5-147)
Supplement: Additional File 2 — The original results of the above study (non-essential files are deleted to keep the file size under the limit set by BMC bioinformatics). [file 1471-2105-5-147-S2.bz2 › chilibotAdditionalFile2/dip10/422ID11470772E10351/html/CCL16_CCR1.html]

 


 **CCL16** and **CCR1** 
  
Found 20 abstracts in PubMed, retrieved 10.  
 

 What does Google say? 
 PDF only 
| .edu only 

---

**Interactive relationship** (e.g. stimulation, inhibition, etc)

**Neutral relationship**- Human CC chemokine liver expressed chemokine  [ **CCL16** ]   **CCL16**  is a functional ligand for  **CCR1** , CCR2 and CCR5, and constitutively expressed by hepatocytes.  Ref: 11470772 Int Immunol, 2001
- Consistently, LEC  [ **CCL16** ]  was found to bind to  **CCR1** , CCR2 and CCR5 with relatively low affinities.  Ref: 11470772 Int Immunol, 2001
- At relatively high concentrations, LEC  [ **CCL16** ]  induced calcium mobilization and chemotaxis via  **CCR1**  and CCR2.  Ref: 11470772 Int Immunol, 2001
- Taken together, LEC  [ **CCL16** ]  is a new low affinity functional ligand for  **CCR1** , CCR2 and CCR5, and is constitutively expressed by liver parenchymal cells.  Ref: 11470772 Int Immunol, 2001
- LEC  [ **CCL16** ]  has been shown to chemoattract monocytes, lymphocytes, and polymorphonuclear leukocytes PMNs by its binding to  **CCR1**  and CCR8 chemokine receptors.  Ref: 12843794 J Immunother< MedlineTA>J Immunother, 2001
